# Supplementary material for: Splice-Junction-Based Mapping of Alternative Isoforms in the Human Proteome
Source: Cell Rep. Author manuscript; Available in PMC 2020 Jan 15. (PMC6961840; doi:10.1016/j.celrep.2019.11.026)
Supplement: 2 [file NIHMS1546469-supplement-2.zip › DF1/PXD009737/TestisTrypsin/TestisTrypsin_14_GOT2_ISVAGVTSSNVGYLAHAIHQVTR.pdf]

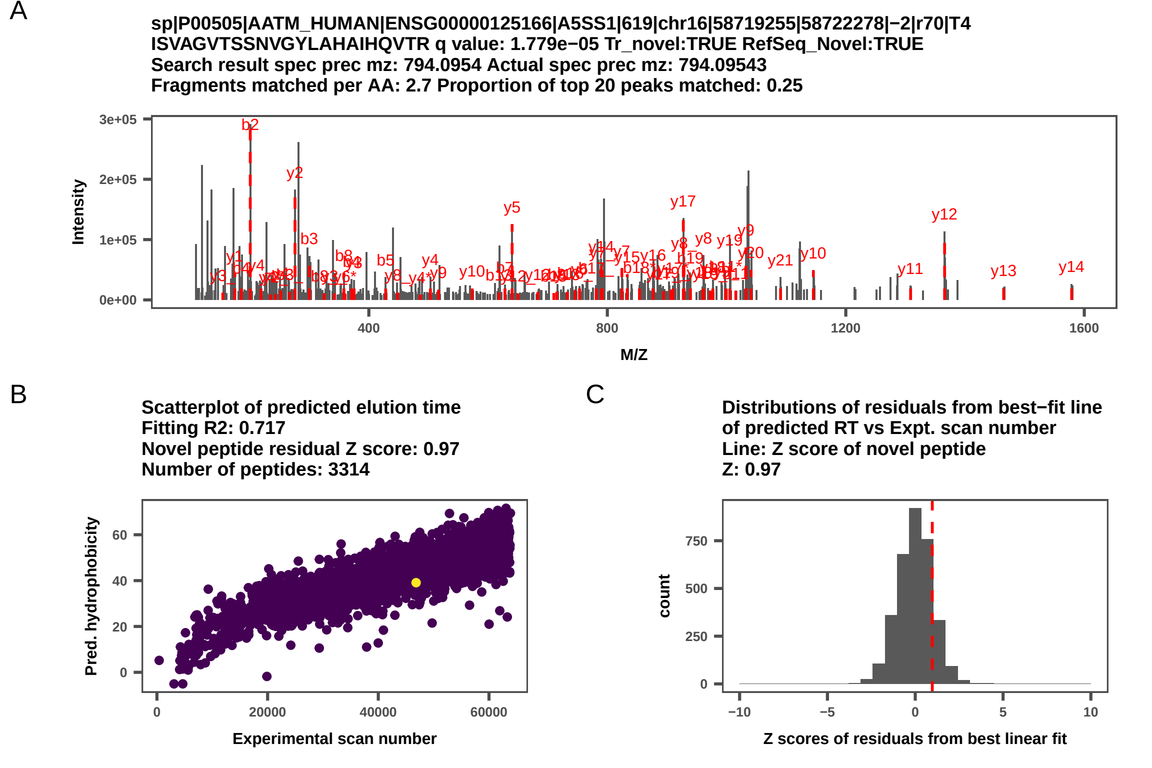
A
sp|P00505|AATM_HUMAN|ENSG00000125166|A5SS1|619|chr16|58719255|58722278|−2|r70|T4
ISVAGVTSSNVGYLAHAIHQVTR q value: 1.779e−05 Tr_novel:TRUE RefSeq_Novel:TRUE
Search result spec prec mz: 794.0954 Actual spec prec mz: 794.09543
Fragments matched per AA: 2.7 Proportion of top 20 peaks matched: 0.25
Intensity
3e+05
2e+05
1e+05
0e+00
b2
y2
b3
y5
y17
y12
y10
y11
y13
y14
y21
y20
b5
y4
M/Z
400
800
1200
1600
B
Scatterplot of predicted elution time
Fitting R2: 0.717
Novel peptide residual Z score: 0.97
Number of peptides: 3314
Pred. hydrophobicity
60
40
20
0
0
20000
40000
60000
Experimental scan number
C
Distributions of residuals from best−fit line
of predicted RT vs Expt. scan number
Line: Z score of novel peptide
Z: 0.97
count
750
500
250
0
−10
−5
0
5
10
Z scores of residuals from best linear fit
